# Middle East Respiratory Syndrome Coronavirus Intra-Host Populations Are Characterized by Numerous High Frequency Variants

**DOI:** 10.1371/journal.pone.0146251

**Published:** 2016-01-20

**Authors:** Monica K. Borucki, Victoria Lao, Mona Hwang, Shea Gardner, Danielle Adney, Vincent Munster, Richard Bowen, Jonathan E. Allen

**Affiliations:** 1 Lawrence Livermore National Laboratory, Livermore, California, United States of America; 2 Colorado State University, Fort Collins, Colorado, United States of America; 3 National Institutes of Health, Rocky Mountain Laboratories, Hamilton, Montana, United States of America; German Primate Center, GERMANY

## Abstract

Middle East respiratory syndrome coronavirus (MERS-CoV) is an emerging human pathogen related to SARS virus. *In vitro* studies indicate this virus may have a broad host range suggesting an increased pandemic potential. Genetic and epidemiological evidence indicate camels serve as a reservoir for MERS virus but the mechanism of cross species transmission is unclear and many questions remain regarding the susceptibility of humans to infection. Deep sequencing data was obtained from the nasal samples of three camels that had been experimentally infected with a human MERS-CoV isolate. A majority of the genome was covered and average coverage was greater than 12,000x depth. Although only 5 mutations were detected in the consensus sequences, 473 intrahost single nucleotide variants were identified. Many of these variants were present at high frequencies and could potentially influence viral phenotype and the sensitivity of detection assays that target these regions for primer or probe binding.

## Introduction

Middle East respiratory syndrome coronavirus (MERS-CoV) is an emergent beta coronavirus related to SARS virus, and is capable of causing severe respiratory symptoms in humans. Since the discovery of MERS-CoV in 2012, MERS-CoV infections have been detected in 23 countries with local transmission occurring in over half of the affected countries [1]. Although the mortality rate is high (about 40%), subclinical infections may be more prevalent than severe disease, which occurs mainly in elderly individuals and those with pre-existing conditions. Serologic, genetic, epidemiological, and animal infection studies indicate that dromedary camels (DC; *Camelus dromedarius*) serve as a reservoir for human infection [2–4]. Seropositive assay results have been obtained from DC samples collected throughout the Arabian Peninsula and parts of Africa, including regions where no human cases have been detected [5], however, assay results may be impacted by the presence of cross reactive coronavirus antibodies [1]. Experimental infection of DCs resulted in a self-limiting upper respiratory infection with viral shedding occurring primarily through nasal secretions [2].

MERS-CoV is a member of *Betacoronavirus* genus lineage 2c [6] and has a genome of positive-sense RNA 30,119 nt in length [7,8]. The first two thirds of the genome codes for a replicase polyprotein, ORF1ab, which contains multiple nonstructural proteins nested within the two open reading frames (ORF1a and ORF1b). The 3’ end of the genome codes for structural proteins: spike glycoprotein, envelope, membrane, and nucleoprotein, and five nonstructural proteins, ORFs 3, 4a, 4b, 5, and 8b [7].

Illumina sequencing of the MERS genome has indicated the presence of high frequency variants in intrahost viral populations [9–11]. To characterize the genetic variants present in nasal swabs from camels experimentally infected with a low passage human MERS-CoV isolate, we amplified a majority of the MERS genome using multiplexed RT-PCR assays and sequenced the products at high depths of coverage. The data generated from this analysis indicate that high frequency intrahost single nucleotide variants (iSNVs) occur throughout the genome and suggest that variant genotypes are present at high enough frequency to be readily transmitted and potentially to impact viral phenotype.

## Materials and Methods

### Virus

MERS-infected nasal samples were derived from animal work which was previously approved by the Institutional Animal Care and Use Committee of Colorado State University and was performed in compliance with recommendations in the Guide for the Care and Use of Laboratory Animals of the National Institute of Health with every effort to minimize animal suffering [2]. Nasal swabs were collected on days 1, 3 and 5 post-infection from three camels experimentally infected at Colorado State University with MERS-CoV strain HCoV-EMC/2012, as described in Adney et al., 2014 [2]. The viral stock used for the infections was provided with the permission of the Department of Viroscience, Erasmus Medical Center, Rotterdam, The Netherlands and had been propagated in Vero E6 cells. Vero passage 7 of HCoV-EMC/2012 (“Seed”) was used for the camel infections and served as the pre-*in vivo* passage tissue culture control. No additional cell culture passages were performed as a control for the subsequent passage of the virus from Vero cells into camels. MERS-CoV was obtained from BEI Resources (EMC/2012, NR-44260) for generation of viral cDNA for testing of PCR primers prior to the arrival of nasal samples and seed stock. Note, the number assigned to each camel in this study differ from those assigned to the same camels in Adney et al. [2] (work performed at Colorado State University (CSU)). Camel 1 from this study corresponds to CSU Camel 2, Camel 2 corresponds to CSU Camel 3, and Camel 3 corresponds to CSU Camel 1.

### Primer design

Primers were designed that spanned a majority of the genome yielding RT-PCR products of 2–3 kb which overlapped by approximately 100 nt (S1 Table, S1 Fig). With the exception of the first primer set, each region was covered by two primer sets. The primers were designed to perform as two highly multiplexed reactions as described in Gardner et al., 2014 [12].

### RNA extraction and RT-PCR

Total RNA was extracted using TRIzol^®^ LS Reagent (Invitrogen) following the manufacturer’s protocol. Reverse transcription was performed using random hexamers and the Superscript III RT reverse transcriptase kit (Invitrogen). Viral cDNA templates were amplified using Q5^®^ Hot Start High-Fidelity 2X Master Mix (New England BioLabs, MA), following manufacturer's instructions. PCR conditions consisted of 98°C for 30 s, followed by 35 cycles of 98°C for 10 s, 60°C for 20 s, and 72°C for 1 min. The final cycle was 72°C for 2 min. PCR products were prepared for sequencing using the QIAquick PCR Purification kit (Qiagen, CA).

### Illumina sequencing

Samples were sequenced using 150 paired end reads in multiplex (4 lanes) using the Illumina HiSeq2500's Rapid Run Mode at the Vincent J. Coates Genomics Sequencing Laboratory at the University of California, Berkeley. A plasmid control was used to determine the error rate of the PCR and Illumina sequencing as described previously [13].

### Computational analysis

The methods used for analysis of the Illumina data is described in detail in our previous publication [13]. The open source read mapping software SHRiMP2, which was shown to have high read mapping sensitivity was chosen for the tool’s ability to map as many reads as possible in the face of individual errors within each read [14,15]. Variants showing up in 2 or more samples and for which there was at least 100 overlapping read pairs (ORP) were detected in each sample, and were detected at 473 positions in the genome; further analysis was prioritized for these sites. All primers were screened against the reads to trim primer sequences at the ends of reads using fastq-mcf [16] and default parameters. Genomic regions overlapping primer regions were examined separately to consider the potential for un-trimmed primer errors to remain in reads mapped to the reference genome. Consensus sequence that yielded the highest quality data for each camel was submitted to GenBank along with the Seed consensus sequence. The Illumina reads were submitted to the GenBank Sequence Read Archive (BioProject number PRJNA302555).

## Results and Discussion

### Genome amplification

**Fig 1:**
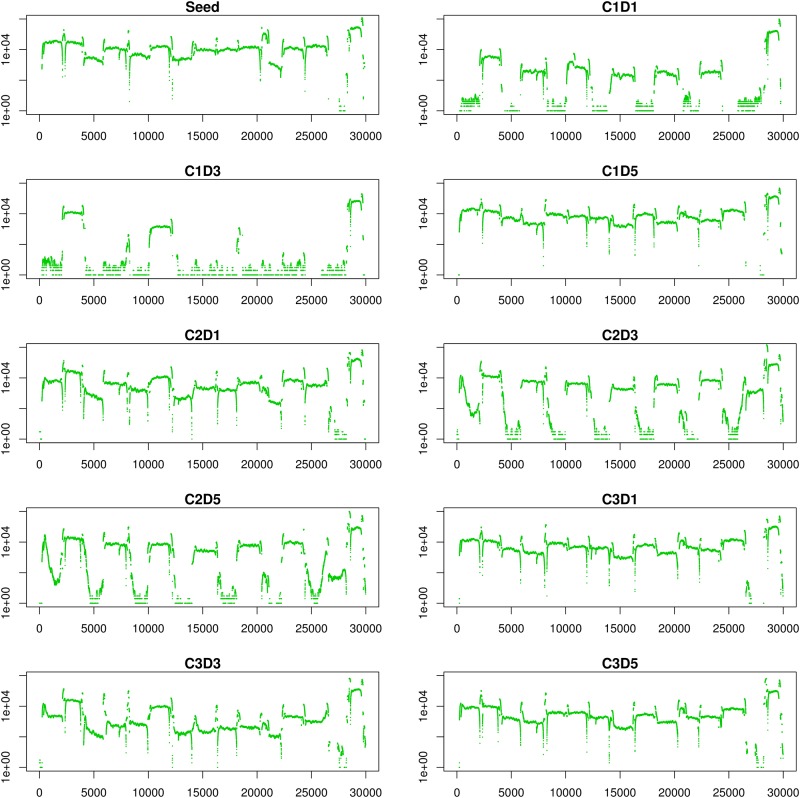
Coverage Plots. UDS coverage depth across the genome was plotted for each sample. The X axis shows the genome position and the Y axis shows the depth coverage (log scale).

The PCR primers designed to span the MERS genome coding regions were tested individually using cDNA derived from the MERS-CoV stock obtained from BEI Resources (EMC/2012, NR-44260). All primer pairs yielded product of correct size (S1 Fig). Primers were then tested in staggered sets divided in two multiplex reactions and the presence of each primer set product was accessed using nested PCR of primer overlap regions as described in Gardner et al., 2014 (S1 Fig) [12]. Discrepancies in overlap product sizes were noted in some primer sets covering the last 4000 nts of the genome. These products were sequenced via Sanger sequencing and this confirmed that the primers bound to and amplified the correct area of the genome, however in some cases the amplified sequence terminated unexpectedly resulting in a truncated sequence output. A third multiplex reaction was generated that contained primers to span this region and in some cases this enabled a complete amplification of this region (Fig 1).

### Data quality and coverage

S2 Table shows coverage for each iSNV site for which variation was detected in two or more samples. The coverage and read depth varied between time points and camels. Camel 2 day 3 (C2D3) provided the most sequence data with an average read depth of 15,378 x, and 28,488 nt sites covered by at least 100 ORP. C1D5 provided the most complete data for Camel 1 with an average read depth of 15,123 and 28,057 nt sites covered. All time points collected from Camel 3 yielded fairly complete data with C3D1 providing the highest average coverage at 12,585 x depth and with 28,393 nt sites covered. Average coverage for the Seed stock was 29,454 x and 28,075 nt positions were covered.

The sequence analysis conducted in this study could not explicitly differentiate between genomes derived from packaged or unpackaged sources. Thus some of the detected mutations could originate from viral genomes that are unable to replicate and persist in isolation. To reduce the emphasis of mutations associated with limited functional relevance, the analysis focused on iSNVs found in high abundance and/or present in two or more samples, and because multiple time points (days 1, 3, and 5) were analyzed for each camel, our analyses favored genomes that persisted during the infection. The expectation is that even when a viral genome is unable to replicate independent of the surrounding population, its presence at sufficient abundance could imply a functional role in the evolution of viral quasispecies. For example, it has previously been shown that defective genomes may function as “defective interfering particles” and may impact viral infection dynamics such as viral persistence [17–19]. Nevertheless, it is important to consider that mutations that are observed at ultra-low abundance and occur rarely could represent viruses with limited functional impact.

### Consensus sequence changes

**Fig 2:**
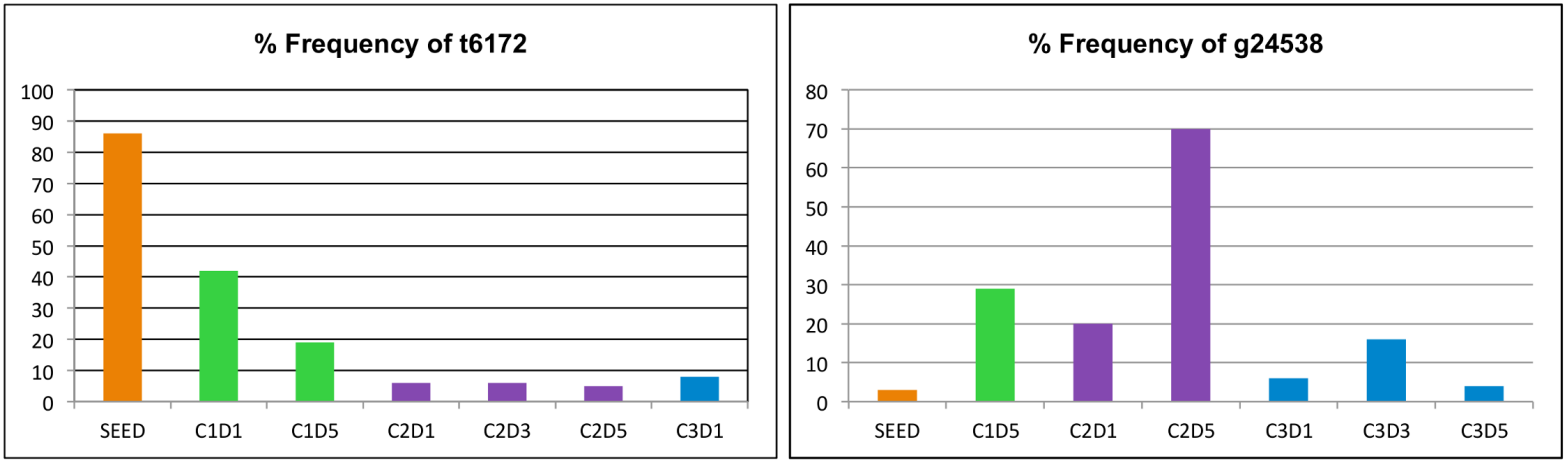
Histogram of high frequency iSNVs resulting in a nonsynonymous change in the consensus sequence of one or more samples. The iSNV detected at site t6172, caused a Phe to Ser mutation in nsp 3 residue 1965 and resulted in a consensus change that differentiated the camel genotypes from that of the Seed. An iSNV, g24538, was present at low frequency in the Seed stock but increased in frequency during propagation in camels with a change in consensus sequence occurring in C2D5, resulting in an N1028S mutation. Note, only data from samples with adequate coverage at the relevant iSNV position were included in the histogram.

Despite the presence of many high frequency iSNVs, change in the consensus sequence was rare. Two different iSNVs were detected at nt 6172 of nsp3 resulting in two different amino acids, Phe and Ser, at residue 1965. The Seed virus had a C nt present in 14% of the reads with T as consensus coding for Phe in 86% of reads. Six of the 9 camel samples had a T iSNV in 5–42% of reads and a C as consensus (S2 Table, Fig 2). The residue change found in the camel samples, F1965S, and was particularly prevalent in each of the two samples from Camel 1 (C1) for which there was coverage at this site; C1 day 1 (C1D1) had 42% and C1D5 had 19%. The percent of reads with the T iSNV declined between day 1 and 5 for all samples (C1- 42% to 19% (day 1 to day 5), C2- 6%, 6%, and 4.5%, days 1–3 respectively, and C3 had 8% day 1 with none detected on day 5 (coverage >1000x, S2 Table).

Synonymous consensus changes were detected; a8396c occurred in the C2D5 sample (80.5% of reads), and t24059c occurred in the spike protein ORF and this change was associated with high frequency variants in many of the samples. The consensus in the Seed sample was a T which was present in 88% of reads and a C present as a variant in 12% of reads. All camel samples had C as consensus at nt 24059. Five of the 9 camel samples yielded sufficient data to detect variant reads, and the presence of a T iSNV was detected in 4 out of 5 samples at frequencies ranging from 3% to 36%. The apparent selection for this nt change in most of the camel samples is interesting given that it should not impact amino acid sequence or phenotype.

A change in consensus sequence occurred at nt 24499 and resulted in an amino acid change, N1015T. The A to C transversion was evident in the deep sequence variant genotypes as well. Seed sequence data had the consensus nt, A, present at 88.5% and a C present at 11.5%. Seven of the 9 camel sequences had coverage at this site and the consensus for these sequences was C, however 2 of the 7 sequences had an A present as a variant at 17.6 and 3.0% for C1D5 and C3D1 respectively. The N1015T mutation detected may be a response to the change from cell culture to an *in vivo* environment. Scobey et al. recently demonstrated that a T1015N mutation resulted from serial tissue culture passage and increased *in vitro* fitness and plaque size [20], thus it seems likely that the N residue has reduced fitness *in vivo* thus resulting in the incremental decrease in frequency seen in the sequence data from these 2 camels.

A consensus change at nt 24538 was detected in C2D5 reads resulting in an N1028S mutation (Table 1, Fig 2). This position had relatively low coverage, 344x depth, and the iSNV (a24538g) was detected at 70%. High frequency iSNVs were also detected at residue nt 24538 with frequencies between 4–30% in all camel samples that have >350x coverage, and at 3% in the Seed stock. This site is one of the several HR1 residues that form hydrogen bonds with HR2 and are part of the hydrophilic interactions between the domains that stabilize binding of the domains [21].

**Table 1:** Nonsynonymous mutations occurring at >1% at sites with at least 100 X ORP coverage.

Nt pos.	Residue	Data type	SEED		C1D1		C1D3		C1D5		C2D1		C2D3		C2D5		C3D1		C3D3		C3D5		ORF
2169	I631L	Consensus	A	0.985	A	1.000	A	0.926	A	0.938	A	0.928	A	0.894	A	0.931	A	0.964	A	0.877	A	0.962	nsp2
		iSNV	C	0.015	NA		C	0.074	C	0.062	C	0.072	C	0.106	C	0.069	C	0.036	C	0.123	C	0.038	
3475	K1066T	Consensus	A	1.000	A	1.000	A	1.000	A	1.000	A	1.000	A	1.000	A	0.992	A	1.000	A	1.000	A	0.804	nsp3
		iSNV	0		NA		NA		NA		0		NA		C	0.009	NA		0		C	0.197	
6161	L1961V	Consensus	G	1.000	G	0.993	0		G	1.000	G	1.000	G	1.000	G	1.000	G	1.000	G	1.000	G	0.892	
		iSNV	NA		T	0.008	NA		NA		NA		NA		NA		NA		NA		C	0.108	
6172	F1965S	Consensus	T	0.858	C	0.583	0		C	0.812	C	0.938	C	0.939	C	0.955	C	0.921	C	1.000	C	1.000	
		iSNV	C	0.142	T	0.417	NA		T	0.188	T	0.062	T	0.061	T	0.045	T	0.079	NA		NA		
8050	D2591A	Consensus	A	0.941	A	1.000	A	1.000	A	1.000	A	0.622	A	0.998	A	0.994	A	0.912	A	0.923	A	0.922	
		iSNV	C	0.060	NA		NA		NA		C	0.378	C	0.002	C	0.006	C	0.088	C	0.077	C	0.078	
8614	M2779T	Consensus	T	1.000	0		0		T	0.942	T	0.845	0		0		T	0.951	T	1.000	T	0.850	nsp4
		iSNV	0		NA		NA		C	0.058	C	0.155	NA		NA		C	0.050	NA		C	0.150	
9798	D3174H	Consensus	G	0.998	0		0		G	1.000	G	0.896	0		0		G	1.000	G	1.000	G	1.000	
		iSNV	A	0.002	NA		NA		NA		C	0.104	NA		NA		NA		NA		NA		
13234	E4319V	Consensus	A	1.000	0		0		A	1.000	A	1.000	0		0		A	0.855	A	1.000	A	0.940	nsp10
		iSNV	0		NA		NA		0		NA		NA		NA		T	0.145	NA		T	0.060	
23150	E565D	Consensus	G	1.000	G	1.000	0		G	1.000	G	0.884	G	0.948	G	0.877	G	0.814	G	0.875	G	0.931	S gene
		iSNV	0		NA		NA		0		T	0.116	T	0.052	T	0.123	T	0.186	T	0.125	T	0.069	
24474	A1007T	Consensus	G	1.000	0		0		G	0.986	G	0.721	G	1.000	G	0.969	G	0.820	G	0.821	G	0.889	
		iSNV	NA		NA		NA		A	0.014	A	0.279	NA		A	0.031	A	0.180	A	0.179	A	0.111	
24499	N1015T	Consensus	A	0.886	0		0		C	0.824	C	0.959	C	1.000	C	1.000	C	0.970	C	1.000	C	1.000	
		iSNV	C	0.115	NA		NA		A	0.176	A	0.041	NA		NA		A	0.030	NA		NA		
24502	N1016S	Consensus	A	0.986	0		0		A	0.967	A	1.000	A	1.000	A	1.000	A	0.956	A	1.000	A	0.978	
		iSNV	G	0.014	NA		NA		G	0.033	NA		NA		NA		G	0.044	NA		G	0.022	
24538	N1028S	Consensus	A	0.966	0		0		A	0.710	A	0.803	A	1.000	G	0.698	A	0.944	A	0.845	A	0.956	
		iSNV	G	0.034	NA		NA		G	0.290	G	0.197	NA		A	0.302	G	0.056	G	0.155	G	0.044	
27162	Y86*	Consensus	0		0		0		0		0		G	0.878	G	0.944	0		0		0		orf5
		iSNV	NA		NA		NA		NA		NA		A	0.122	A	0.056	NA		NA		NA		
28464	R204S	Consensus	G	1.000	G	0.638	G	0.865	G	0.995	G	0.998	G	0.999	G	0.999	G	0.999	G	0.997	G	0.980	M gene
		iSNV	NA		T	0.362	T	0.100	T	0.005	T	0.001	T	0.001	T	0.001	T	0.001	T	0.002	T	0.020	
28466	S2051	Consensus	G	0.999	G	1.000	G	0.920	G	0.996	G	0.997	G	0.999	G	0.999	G	0.998	G	0.997	G	0.965	
		iSNV	NA		NA		T	0.080	T	0.004	T	0.002	T	0.001	T	0.001	T	0.002	T	0.002	T	0.025	
28587	R8C	Consensus	C	0.993	C	0.996	C	0.993	C	0.990	C	0.996	C	0.950	C	0.996	C	1.000	C	1.000	C	1.000	N gene
		iSNV	T	0.007	A	0.004	T	0.007	T	0.010	T	0.004	T	0.050	T	0.004	NA		NA		NA		
28778	L16P	Consensus	T	1.000	T	1.000	T	1.000	T	1.000	T	0.992	T	1.000	T	0.981	T	0.982	T	0.996	T	0.995	
		iSNV	0		0		0		0		C	0.008	0		C	0.020	C	0.018	C	0.004	C	0.005	
29734	G390V	Consensus	G	1.000	G	1.000	G	0.571	G	0.997	G	0.935	G	0.990	G	0.990	G	1.000	G	1.000	G	0.943	
		iSNV	NA		NA		T	0.429	C	0.003	T	0.065	A	0.010	A	0.005	NA		NA		T	0.057	
29737	S391I	Consensus	G	0.999	G	1.000	G	1.000	G	0.928	G	0.906	G	0.924	G	1.000	G	0.932	G	1.000	G	0.904	
		iSNV	C	0.001	NA		NA		T	0.072	T	0.094	T	0.076	NA		T	0.063	NA		T	0.089	
29747	Q394H	Consensus	G	0.992	0		G	1.000	G	0.985	G	0.936	G	0.800	G	1.000	G	0.992	G	0.876	G	0.911	
		iSNV	A	0.008	NA		NA		NA		T	0.064	T	0.190	NA		A	0.008	T	0.124	T	0.073	
29748	R395C	Consensus	C	0.950	0		C	1.000	C	0.994	C	0.973	C	1.000	C	1.000	C	0.940	C	0.955	C	0.998	
		iSNV	T	0.050	NA		NA		A	0.006	T	0.027	NA		NA		T	0.058	T	0.045	G	0.002	
29782	P406L	Consensus	C	1.000	0		0		0		C	1.000	C	1.000	C	1.000	T	1.000	C	1.000	C	0.787	
		iSNV	0		NA		NA		NA		NA		NA		0		0		NA		T	0.213	
29783	P406L	Consensus	A	1.000	0		0		0		A	1.000	A	1.000	A	1.000	G	1.000	A	1.000	A	0.787	
		iSNV	0		NA		NA		NA		NA		NA		0		0		NA		G	0.213	
29784	M407I	Consensus	A	1.000	0		0		0		A	1.000	A	1.000	A	1.000	T	1.000	A	1.000	A	0.787	
		iSNV	0		NA		NA		NA		NA		NA		0		0		NA		T	0.213	
29788	I408S	Consensus	T	1.000	0		0		0		T	1.000	T	1.000	T	1.000	T	1.000	T	1.000	T	0.858	
		iSNV	NA		NA		NA		NA		NA		NA		NA		NA		NA		G	0.142	
29794	V410S	Consensus	0		0.000		0.000		T	1.000	T	1.000	T	1.000	T	1.000	T	1.000	T	0.917	T	1.000	
29794		iSNV	NA		NA		NA		NA		NA		NA		NA		NA		C	0.083	NA		

A consensus change was detected at nt 29849 for sample C3D1 which had an A present in 66.1% of reads and a G in the remaining 33.9%. All other samples had a G at this site with no variants detected in the 6 samples that had reads covering this site. Nt 29849 is located in the 3’ end of the genome outside of coding regions. This mutation occurs at a nt site within reverse primer NSeq-Rev which is used for PCR amplification and sequencing of the N protein, as described by Corman et al., 2012 [22].

A gap in deep sequence data occurred in the genome region of 26600 to 28300 for samples obtained from Camels 1 and 3, and samples from Camel 2 had relatively low coverage in this region (less than 50x depth) (Fig 1). The reason for this was unclear in that no mutations were detected in the primer regions as determined by sequence data from reads that spanned the primer sites. iSNVs were present and may have impacted sensitivity but no trend was observed between iSNV frequency in primer regions and coverage of in corresponding amplicon regions. The primer set that appears to have failed, primer set 14, had previously yielded product when tested individually (S1 Fig). Lack of extensive deep sequence data for this region is unfortunate given that upE PCR primers designed for MERS CoV detection target this region [19].

### Subconsensus (iSNV) sequence changes

Illumina data obtained from overlapping read pairs (ORPs) was used to define the subconsensus viral populations present in each sample. iSNVs were detected in 473 non-primer positions of the genome at various frequencies. The iSNVs analyzed were limited to those present in two or more samples and for which there was at least 100 ORPs. Sequencing coverage is a key factor in iSNV detection sensitivity since deeper coverage generally increases the number of ultra-rare variants detected. When comparing iSNVs across samples, for each genome position, the least frequent variant detected across all samples sets the maximum sensitivity threshold. Samples were checked for their coverage and a binomial model (p<0.01) was used to determine whether a rare variant should be detected in a sample with the available coverage. When coverage is found to be too low, it implies that the lack of detecting a rare variant cannot be used to infer the lack of a true rare variant present and the position is marked with a “NA” in S1 Table.

#### ORF1ab

The Seed stock had iSNVs present at nts 307–313, part of the nsp1 ORF (residues 9–11). All iSNVs were detected at 4% indicating there is a second genotype present at 4% (Table 1). Six of the 8 camel samples had high coverage at these sites thus it appears that these iSNVs do not survive when transmitted to camels.

**Fig 3:**
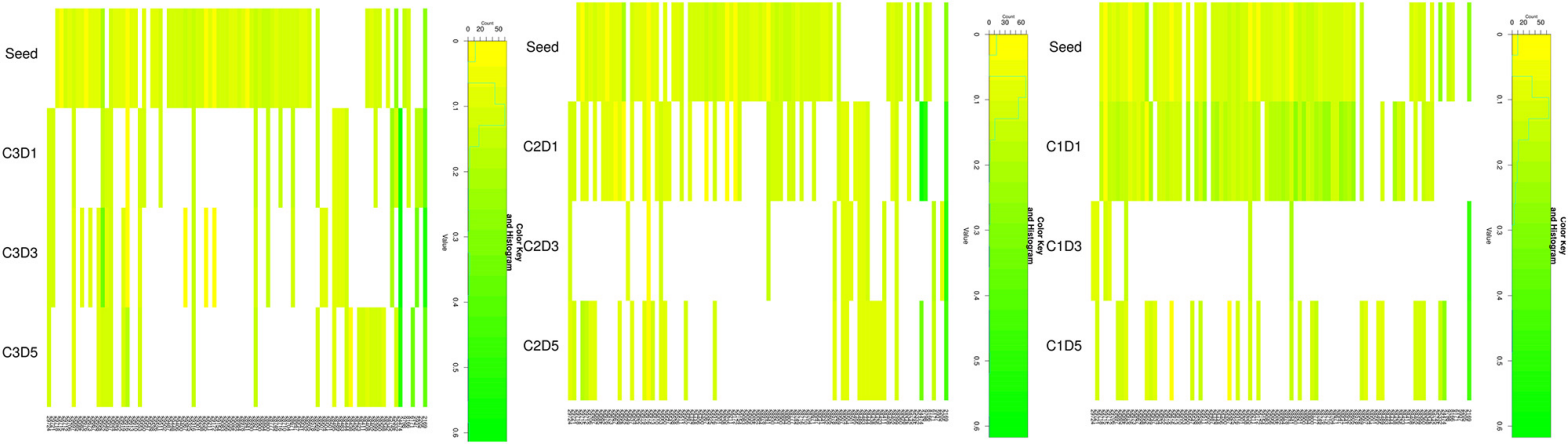
Heat maps of nonsynonymous iSNV frequencies and nt positions. Heat maps for each camel (A-C) show the frequencies and positions of iSNVs for each camel and time point, and for Seed inoculum. The number (count) of iSNV for each frequency value is shown along with the heat map color key is shown above each camel heatmap.

Two nonsynonymous iSNVs were detected in the nsp2 ORF, at nt 2169 residue 631 Ile was replaced by Leu which was present at 2% in Seed and between 4–12% in camel samples (Table 1, Fig 3). At residue 658 (nt 2251) Cys is replaced by Ser in 11% of Seed sequences, whereas the camel samples have this iSNV present 1% or less despite ample coverage. Interestingly, an I631L mutation was noted in strain MERS-CoV Hu/England-N1/2012 [23], a strain that replicated more slowly in Calu-3 cells compared to EMC/2012 (JX869059) strain [24].

All nonsynonymous iSNVs detected in the nsp3 ORF occurred outside of the papain-like protease (PLpro) domain (residues 1476–1804). A high frequency iSNV was detected in 20% of the reads from C3D5 at nt 3475 resulting in a change in nsp3 residue 1066 from Lys to Thr. This iSNV was also detected in C2D5 at 1%, but not in the Seed virus. Coverage in all samples ranged from 3312x (C1D1) to 26,316x (Seed).

Sample C3D5 had adjacent iSNVs in nsp3, both detected at 11% at nts 6159 and 6161 causing a nonsynonymous change, L1961V. The final nonsynonymous iSNV detected in nsp3 occurred at nt 8050, residue D2591A; present in sample C2D1 at 38%, C3D3 and C3D5 at 8%, and Seed at 6%.

The nsp4 ORF had two nonsynonymous iSNVs present in a subset of the camel samples. The first iSNV, nt 8614, resulted in an amino acid change, M2779T, and was present in 4 camel samples at 6–16%. One or more samples from each camel had this iSNV but it was not detected in the Seed sequence. Sequence data was available for 5 of the 9 camel samples, and 4 of the 5 camels had coverage >1000x; the fifth camel sample had coverage of 841x and the Seed sample had coverage of 4289x (S2 Table). A Blastp search showed residue M2779 as conserved in MERS sequences. Additionally, a nonsynonymous iSNV was detected at nt 9798, D3174H, at 10% for C2D1 only. Coverage was similar to that of nt 8614 and a Blastp showed this residue to be conserved.

One nonsynonymous iSNVs was detected in the nsp10 ORF at nt 13234. Amino acid change in the variant genotype, E4319V, was detected in C3D1 and C3D5, at 15% and 6% respectively, although analysis was limited to 5 camel samples due to limited coverage at this site. nsp10 tends to be well conserved among coronaviruses and the mutation occurs in a region which could potentially interfere with viral replication via nsp10-nsp14 interactions [25].

nsp14 had an iSNV detected at nt 19468, resulting in amino acid change R2012S in 22% of reads for Seed only. Six of the 9 camel samples had coverage >1000x indicating that this iSNV was selected against once introduced to the camel host environment. Although nsp14 5’end encodes a 3′-to-5′ exoribonuclease (ExoN), this iSNV occurred in a domain within nsp14 encoding a (guanine-N7)-methyltransferase [26,27].

#### Spike gene

Two consensus changes were detected in the spike ORF, nt t24059c and nt a24499c, which result in a nonsynonymous mutation of N1015T. High frequency iSNVs were detected at both of these sites in addition to other iSNVs which were not associated with a consensus change.

Two regions of the spike protein had high frequency nonsynonymous iSNVs present- the RBD and the HR1 domain of the S2 subunit (residues 998–1039) which has a role in fusion [21]. The iSNV which occurred in residue 565 of RBD occurred in a region identified as the patch 2 region (RBM; amino acids 484–567) that interfaces with the host receptor hDPP4 [21]. This iSNV, nt t23150, residue E565D, was present in 3/3 samples from Camels 2 and 3, at a frequency of 5–20% (Table 1) but was not detected in the Seed sample. This residue however, has not been shown to be associated with any defined escape mutations [28].

High frequency nonsynonymous iSNVs were detected at 3 sites in the HR1 domain- A1007T, N1015T, and N1028S, and lower frequency iSNVs (<5%) were detected at residue 1016. High frequency iSNVs coding for a nonsynonymous mutation, A1007T, in the HR1 domain were detected at nt 24474. C2D1 had an adenine present at 28%, and C3D1 and D3 had this iSNV at 18%, and D3 at 11%. Two of the three camel samples with no iSNV detected had relatively low coverage (71x and 387x; S2 Table). There were no iSNVs detected at this site in the Seed data although coverage at this site was fairly good (4313x).

Nts 24499 and 24502 had iSNVs present that effected adjacent residues (residues N1015T, N1016S). Four of the 9 camel samples had >1000x coverage at these sites, and two of these samples had iSNVs. C1D5 had iSNVs at both sites at a frequency of 18% for C24499A and 3% for A24502G. C3D1 had iSNVs present at 3% and 4%, and the Seed had 12% and 1% for these sites, respectively.

#### ORF5

An iSNV resulting in a stop codon at residue 109 was detected in 12% of reads at nt 27162 for camel C2D5, the only sample for which there was adequate coverage at this site (>100x, S2 Table). This would truncate the protein roughly in half. This iSNV was also detected in 45% of reads for this strain of MERS in a previous study and maybe an artifact of passage in cell culture [7].

#### M gene

A high frequency iSNV was detected in the M protein ORF, nt 28464, resulting in amino acid change R204S. In particular, Camel 1 had the mutation detected in 36% of reads on day 1, 10% of reads on day 2 and 1% on day 3. Sample C3D5 had the mutation in 2% of reads. Adjacent residue, 205, was also variable with Ser mutating to Ile in 8% of reads for C1D3 and 3% of reads for C3D5. Given the proximity of the two reads one would expect the mutations to be linked, thus the lack of variation for C1D1 residue 205 was surprising. Sequence coverage was excellent for all samples at this site and no variation was detected in the Seed data. These mutations occur in a region that may impact the interaction of the M protein with the N protein [29].

#### N gene

All nonsynonymous changes occurred in the intrinsically disordered regions (IDR) of the nucleoprotein ORF. IDRs have been shown to modulate RNA binding and oligomerization of the N protein [30–32], display exceptional genetic plasticity [33], and may play a role in cross-species transmission of zoonotic coronaviruses including MERS-CoV [34,35].

Two of the 13 residue mutations occurred in the 5’ IDR and occurred in limited number of samples. The iSNV at nt 28587, R8C, was detected only in C2D3 in 5% of the reads and in the Seed at 1%. A second residue variant was detected at nt 28778, L16P, in 5 of the 9 camel samples at between 0.4 and 2.0%, but was not detected in the Seed despite excellent coverage (>218K).

The remaining nonsynonymous iSNVs occurred in the IDR at the 3’ end of the ORF, and some were detected at high frequency. In particular, a cluster of iSNVs were detected between nts 29734 and 29748 (residues 390–395) despite rather limited coverage for this region. For example, at G390V mutation was detected at 43% in C1D3 reads (nt 29734), and at 7% for C2D1, and 6% for C3D5, but not in the Seed data. Coverage was limited, less than 5K depth for all samples. An iSNV was detected at nt 29736, S391R, at 8% for the Seed sample. Amino acid change S391I was detected in 6–9% for five camel samples (C1D5, C2D1, C2D3, C3D1, C3D5) but not in the Seed data. Similar to previous sites, coverage was relatively low. Three adjacent iSNVs were detected at between 4–10% in the Seed data at nts 29739–41 resulting in I392F. A variant was detected at nt 29743 which resulted in a Thr to Ile mutation at residue 392 in 4% of reads from C2D5. The same residue was present as a Val variant for the Seed data due to two adjacent iSNVs at 29742 and 29743 present at 4% and 7% respectively.

An iSNV at nt 29747 was detected in several camel samples and resulted in a nonsynonymous change, Q394H. The iSNV was detected in reads from both C2D1 and C2D3 at 6% and 19% respectively, and also in C3D3 and C3D5 at 12% and 7% respectively. Lack of detection in C1 data may have been due to very low coverage at this site for D1 and D3 (coverage 42 and 284 respectively), however the Seed coverage was considerably greater, 3424x, indicating that if the iSNV was present in the Seed inoculum it was relatively rare and this positively-charged variant may have been arisen due to the change in host environment (*in vitro* to *in vivo*). A search of GenBank data revealed that H394 is not represented in any of the MERS sequence data available, although the corresponding residue for other betacoronaviruses MHV and HCoV-OC43 is positively charged (Arg).

Multiple camel samples had an iSNV at nt 29748 resulting in a R395C occurring at a frequency of 3% for C2D1, 6% for C3D1, and 5% for C3D3, although coverage was limited as described above. Interestingly a different iSNV was present in the Seed reads at this site, R395Y, in 5% of reads. These data indicate that this region is a hot spot for genetic diversity, and notably there is no indication that these iSNVs are linked given that they vary in frequency and are often present in different samples.

C3D5 had a unique set of high frequency variants in this region caused by 3 consecutive nt changes (29782–84) resulting in 2 amino acid changes, P406L and M407I, both of which were detected at 21%. The adjacent nts 29788 and 29794 were the site of 2 more nonsynonymous changes detected in C3D5 data; I408S and V410A, present at 14% and 8% respectively. It is notable that the other camel samples and the Seed sample all had very high coverage in this region, ranging from 50K-220K in depth.

The presence of numerous high frequency variants in the 3’ end of the N protein, well past the end of the ORF8b coding region (nt 29100) underscores the potential genetic diversity of this virus. A study by Agnihothram et al., 2014 revealed that a human strain of MERS-CoV, Hu/England-N1/2012 had 2 amino acid deletions in this region of the N protein, residues 391 and 392 [24].

### Insertion and deletion (Indel) variant data

Indels detected at >1% were analyzed to identify hotspots and potential impact. No indels occurred in regions that would affect primer binding. Eight positions were identified that had an insertion present at greater than 1%. Six of the 8 inserts occurred in the nsp3 gene, and all 8 were within ORF1ab. The highest frequency insert, “A”, occurred at nt 6074 and was detected at 5.2% for C3D3 as well being present as an “TTTA” insert for 1.2% of reads for C2D3.

Insertions that were present for all or the majority of camel samples were also found in the Seed data. For example Seed had an “AA” insertion at site 2845 at 0.0029%, and camels 2 and 3, days 1, 3, and 5 had this insert detected at between 0.01 and 0.02%. Interestingly camel 1 had only a single “A” present at this site and it was detected at 1.1% (day 1) to 0.3% (days 3, 5).

Deletions detected at greater than 1% occurred at 21 sites and ranged in frequency from 1.3% to 15.3%, and the number of bases deleted ranged from 1 to 20 nts. The two highest frequency deletions occurred at nt 26856 and were detected in C2D3 at 15.3% and C2D5 at 6.1%; both with 4 nts deleted.

### Nonsynonymous (Ka) and synonymous (Ks) substitution rates

To check for evidence of selective pressure within samples, the Ka/Ks ratio was checked for each gene using one representative passage of each camel (C1D5, C2D1 and C3D5) and the seed stock. Ka/Ks ratios were calculated for each sample using the KaKs calculator [36]. To calculate the ratio for each gene in each sample, the consensus sequence was compared with a concatenation of the rare variants, which are treated as a second sequence. Only one gene in one sample (C2D1, M pro) showed significant divergence from 1 (Ka/Ks = 1.49, p-value < 0.05). Thus, there appeared to be limited evidence for an identifiable selective pressure on the sub-population of each sample.

## Conclusion

Next generation sequencing (NGS) platforms such as Illumina enable viral populations to be defined with high granularity and provide insight into the role of variant genotypes in viral virulence and transmission. Previously NGS was used to rapidly obtain consensus sequences of multiple MERS-CoV samples [9]. Unexpectedly, analysis of the consensus sequence data revealed the presence of MERS-CoV variants at relatively high frequencies, thus leading the authors to speculate about the role of viral quasispecies in cross-species transmission. Results of the present study demonstrate the value of ultra-deep Illumina sequencing for identification of both rare and high frequency variants present in the nasal samples of three camels that had been experimentally infected with a human isolate of MERS-CoV [2].

### Potential impact of high frequency iSNVs on biosurveillance

Infection of camels with a human isolate of MERS-CoV induced numerous high frequency iSNVs throughout the genome upon a single passage in camels. This indicates that MERS-CoV, similar to other coronaviruses, is prone to extreme genetic diversity in intrahost viral populations that may change rapidly in response to a change in host environment [9,11,37–40]. Until patterns of variation are defined for quasispecies present in non-laboratory passaged MERS-CoV samples, design of reagents for detecting MERS-CoV infections will be vulnerable to the effects of intrahost genetic diversity on reagent sensitivity. For example, at nt 2966, there is an iSNV present at 4% for Seed but greater than 20% in samples from all 3 camels indicating that this iSNV may be selected for in camels. Interestingly, an NCBI Blast search of the 60 nt region including this iSNV site revealed no indication of variability at this site for any of the 99 MERS virus sequences that were present on GenBank. This mutation, although present in a high proportion of the intrahost populations, would not be included in Genbank consensus sequences that are used to identify conserved regions for primer design and thus has the potential to interfere with viral detection assays that include this nt position. Indeed, only 5 mutations were detected in the consensus sequences, although 473 intrahost single nucleotide variants were identified. Many of these variants were present at high frequencies and could potentially influence viral phenotype and adversely affect the sensitivity of detection assays that target these regions for primer or probe binding.

## Supporting Information

S1 Fig PCR amplification of MERS-CoV (strain EMC/2012) using primers tested as individual reactions (A) and in two multiplexed reactions (B). (A) Primer sets are listed above each gel product. (B). Multiplex products include bands of expected size, as well as smaller bands and primer dimers.
(PDF)

S1 Table MERS primer sequences and positions. (PDF)

S2 Table Frequency of consensus and iSNV nts, and position coverage based on ORP data (non primer regions). The iSNV data is distributed between two spreadsheets, the first, S2A Table, shows all iSNVs that detected outside of the primer regions, and the second, S3 Table, shows iSNVs occurring in primer regions. iSNVs detected within primer sites were examined separately to consider the potential for un-trimmed primer errors to remain in reads mapped to the reference genome. In total this table consists of approx. 2000 rows of iSNV data and is available on request as an Excel spreadsheet.
(XLSX)

S3 Table Frequency of consensus and iSNV nts, and position coverage based on ORP data (primer regions). (PDF)
