# Correction: Middle East Respiratory Syndrome Coronavirus Intra-Host Populations Are Characterized by Numerous High Frequency Variants

**DOI:** 10.1371/journal.pone.0154424

**Published:** 2016-04-25

**Authors:** Monica K. Borucki, Victoria Lao, Mona Hwang, Shea Gardner, Danielle Adney, Vincent Munster, Richard Bowen, Jonathan E. Allen

In Table 1, the residue for 27162 should be 107, not 109. Please see the corrected Table 1 here.

**Table 1:** Nonsynonymous mutations occurring at >1% at sites with at least 100 X ORP coverage.

Nt pos.	Residue	Data type	SEED		C1D1		C1D3		C1D5		C2D1		C2D3		C2D5		C3D1		C3D3		C3D5		ORF
2169	I631L	Consensus	A	0.985	A	1.000	A	0.926	A	0.938	A	0.928	A	0.894	A	0.931	A	0.964	A	0.877	A	0.962	nsp2
		iSNV	C	0.015	NA		C	0.074	C	0.062	C	0.072	C	0.106	C	0.069	C	0.036	C	0.123	C	0.038	
3475	K1066T	Consensus	A	1.000	A	1.000	A	1.000	A	1.000	A	1.000	A	1.000	A	0.992	A	1.000	A	1.000	A	0.804	nsp3
		iSNV	0		NA		NA		NA		0		NA		C	0.009	NA		0		C	0.197	
6161	L1961V	Consensus	G	1.000	G	0.993	0		G	1.000	G	1.000	G	1.000	G	1.000	G	1.000	G	1.000	G	0.892	
		iSNV	NA		T	0.008	NA		NA		NA		NA		NA		NA		NA		C	0.108	
6172	F1965S	Consensus	T	0.858	C	0.583	0		C	0.812	C	0.938	C	0.939	C	0.955	C	0.921	C	1.000	C	1.000	
		iSNV	C	0.142	T	0.417	NA		T	0.188	T	0.062	T	0.061	T	0.045	T	0.079	NA		NA		
8050	D2591A	Consensus	A	0.941	A	1.000	A	1.000	A	1.000	A	0.622	A	0.998	A	0.994	A	0.912	A	0.923	A	0.922	
		iSNV	C	0.060	NA		NA		NA		C	0.378	C	0.002	C	0.006	C	0.088	C	0.077	C	0.078	
8614	M2779T	Consensus	T	1.000	0		0		T	0.942	T	0.845	0		0		T	0.951	T	1.000	T	0.850	nsp4
		iSNV	0		NA		NA		C	0.058	C	0.155	NA		NA		C	0.050	NA		C	0.150	
9798	D3174H	Consensus	G	0.998	0		0		G	1.000	G	0.896	0		0		G	1.000	G	1.000	G	1.000	
		iSNV	A	0.002	NA		NA		NA		C	0.104	NA		NA		NA		NA		NA		
13234	E4319V	Consensus	A	1.000	0		0		A	1.000	A	1.000	0		0		A	0.855	A	1.000	A	0.940	nsp10
		iSNV	0		NA		NA		0		NA		NA		NA		T	0.145	NA		T	0.060	
23150	E565D	Consensus	G	1.000	G	1.000	0		G	1.000	G	0.884	G	0.948	G	0.877	G	0.814	G	0.875	G	0.931	S gene
		iSNV	0		NA		NA		0		T	0.116	T	0.052	T	0.123	T	0.186	T	0.125	T	0.069	
24474	A1007T	Consensus	G	1.000	0		0		G	0.986	G	0.721	G	1.000	G	0.969	G	0.820	G	0.821	G	0.889	
		iSNV	NA		NA		NA		A	0.014	A	0.279	NA		A	0.031	A	0.180	A	0.179	A	0.111	
24499	N1015T	Consensus	A	0.886	0		0		C	0.824	C	0.959	C	1.000	C	1.000	C	0.970	C	1.000	C	1.000	
		iSNV	C	0.115	NA		NA		A	0.176	A	0.041	NA		NA		A	0.030	NA		NA		
24502	N1016S	Consensus	A	0.986	0		0		A	0.967	A	1.000	A	1.000	A	1.000	A	0.956	A	1.000	A	0.978	
		iSNV	G	0.014	NA		NA		G	0.033	NA		NA		NA		G	0.044	NA		G	0.022	
24538	N1028S	Consensus	A	0.966	0		0		A	0.710	A	0.803	A	1.000	G	0.698	A	0.944	A	0.845	A	0.956	
		iSNV	G	0.034	NA		NA		G	0.290	G	0.197	NA		A	0.302	G	0.056	G	0.155	G	0.044	
27162	Y107*	Consensus	0		0		0		0		0		G	0.878	G	0.944	0		0		0		orf5
		iSNV	NA		NA		NA		NA		NA		A	0.122	A	0.056	NA		NA		NA		
28464	R204S	Consensus	G	1.000	G	0.638	G	0.865	G	0.995	G	0.998	G	0.999	G	0.999	G	0.999	G	0.997	G	0.980	M gene
		iSNV	NA		T	0.362	T	0.100	T	0.005	T	0.001	T	0.001	T	0.001	T	0.001	T	0.002	T	0.020	
28466	S2051	Consensus	G	0.999	G	1.000	G	0.920	G	0.996	G	0.997	G	0.999	G	0.999	G	0.998	G	0.997	G	0.965	
		iSNV	NA		NA		T	0.080	T	0.004	T	0.002	T	0.001	T	0.001	T	0.002	T	0.002	T	0.025	
28587	R8C	Consensus	C	0.993	C	0.996	C	0.993	C	0.990	C	0.996	C	0.950	C	0.996	C	1.000	C	1.000	C	1.000	N gene
		iSNV	T	0.007	A	0.004	T	0.007	T	0.010	T	0.004	T	0.050	T	0.004	NA		NA		NA		
28778	L16P	Consensus	T	1.000	T	1.000	T	1.000	T	1.000	T	0.992	T	1.000	T	0.981	T	0.982	T	0.996	T	0.995	
		iSNV	0		0		0		0		C	0.008	0		C	0.020	C	0.018	C	0.004	C	0.005	
29734	G390V	Consensus	G	1.000	G	1.000	G	0.571	G	0.997	G	0.935	G	0.990	G	0.990	G	1.000	G	1.000	G	0.943	
		iSNV	NA		NA		T	0.429	C	0.003	T	0.065	A	0.010	A	0.005	NA		NA		T	0.057	
29737	S391I	Consensus	G	0.999	G	1.000	G	1.000	G	0.928	G	0.906	G	0.924	G	1.000	G	0.932	G	1.000	G	0.904	
		iSNV	C	0.001	NA		NA		T	0.072	T	0.094	T	0.076	NA		T	0.063	NA		T	0.089	
29747	Q394H	Consensus	G	0.992	0		G	1.000	G	0.985	G	0.936	G	0.800	G	1.000	G	0.992	G	0.876	G	0.911	
		iSNV	A	0.008	NA		NA		NA		T	0.064	T	0.190	NA		A	0.008	T	0.124	T	0.073	
29748	R395C	Consensus	C	0.950	0		C	1.000	C	0.994	C	0.973	C	1.000	C	1.000	C	0.940	C	0.955	C	0.998	
		iSNV	T	0.050	NA		NA		A	0.006	T	0.027	NA		NA		T	0.058	T	0.045	G	0.002	
29782	P406L	Consensus	C	1.000	0		0		0		C	1.000	C	1.000	C	1.000	T	1.000	C	1.000	C	0.787	
		iSNV	0		NA		NA		NA		NA		NA		0		0		NA		T	0.213	
29783	P406L	Consensus	A	1.000	0		0		0		A	1.000	A	1.000	A	1.000	G	1.000	A	1.000	A	0.787	
		iSNV	0		NA		NA		NA		NA		NA		0		0		NA		G	0.213	
29784	M407I	Consensus	A	1.000	0		0		0		A	1.000	A	1.000	A	1.000	T	1.000	A	1.000	A	0.787	
		iSNV	0		NA		NA		NA		NA		NA		0		0		NA		T	0.213	
29788	I408S	Consensus	T	1.000	0		0		0		T	1.000	T	1.000	T	1.000	T	1.000	T	1.000	T	0.858	
		iSNV	NA		NA		NA		NA		NA		NA		NA		NA		NA		G	0.142	
29794	V410S	Consensus	0		0.000		0.000		T	1.000	T	1.000	T	1.000	T	1.000	T	1.000	T	0.917	T	1.000	
29794		iSNV	NA		NA		NA		NA		NA		NA		NA		NA		C	0.083	NA		

There is an incorrect reference to Table 1 in the second sentence of the first paragraph the Results section, “ORF1ab.” The correct sentence is: The Seed stock had iSNVs present at nts 307–313, part of the nsp1 ORF (residues 9–11). All iSNVs were detected at 4% indicating there is a second genotype present at 4%.

The incorrect value reported in Table 1 also appears in the first sentence of the Results section, “ORF5.” The correct sentence is An iSNV resulting in a stop codon at residue 107 was detected in 12% of reads at nt 27162 for camel C2D5, the only sample for which there was adequate coverage at this site (>100x, S2 Table).
