# Supplementary material for: Middle East Respiratory Syndrome Coronavirus Intra-Host Populations Are Characterized by Numerous High Frequency Variants
Source: PLoS One. 2016 Jan 20;11(1):e0146251. doi: 10.1371/journal.pone.0146251 (PMC4720378; doi:10.1371/journal.pone.0146251)
Supplement: S1 Fig — PCR amplification of MERS-CoV (strain EMC/2012) using primers tested as individual reactions (A) and in two multiplexed reactions (B). (A) Primer sets are listed above each gel product. (B). Multiplex products include bands of expected size, as well as smaller bands and primer dimers. (PDF) [file pone.0146251.s001.pdf]

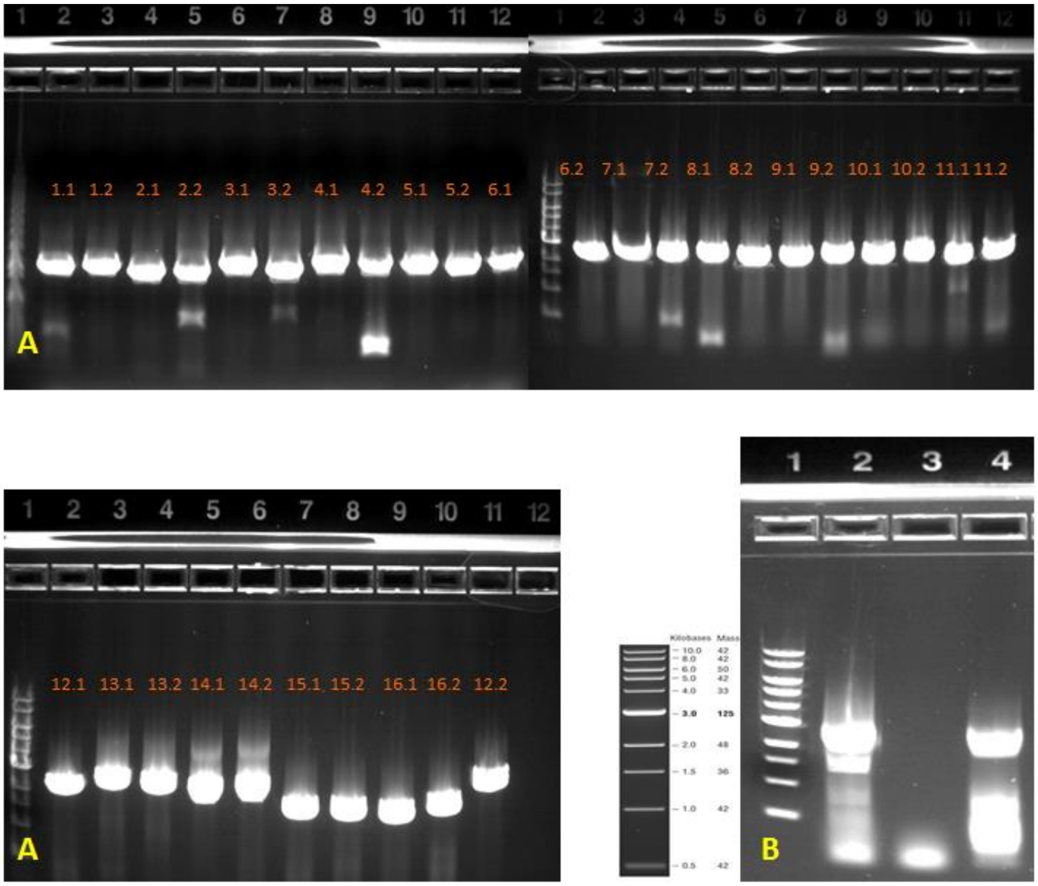

S1 Figure. PCR amplification of MERS-CoV (strain EMC/2012) using primers tested as individual reactions (A) and in two multiplexed reactions (B). (A) Primer sets are listed above each gel product. (B). Multiplex products include bands of expected size, as well as smaller bands and primer dimers.
