# Supplementary material for: Middle East Respiratory Syndrome Coronavirus Intra-Host Populations Are Characterized by Numerous High Frequency Variants
Source: PLoS One. 2016 Jan 20;11(1):e0146251. doi: 10.1371/journal.pone.0146251 (PMC4720378; doi:10.1371/journal.pone.0146251)
Supplement: S1 Table — (PDF) [file pone.0146251.s002.pdf]

S1 Table. MERS primer sequence and positions

Forward Primer Seq	Primer ID	Genome position*		Reverse Primer seq	Primer ID	Genome position*
TTGTACGTCTCGGTCACA	MERS_1F	220		ACTTTGCCATTGACCACCAC	MERS_1.1R	2371
TTATGCTCACAGCGTGTT	MERS_8.1F	2045		CCTGCCGCTGTAGATAAC	MERS_1.2R	2483
GGTGTGTCATCCTTTAGTGAA	MERS_8.2F	2094		ACATCTTGGGAATTAACGACG	MERS_8.1R	4078
AAAAGGGGCTGTCCAAAA	MERS_9.1F	3770		CGCCACGTAACCCATCAT	MERS_8.2R	4146
TCAGCAGGCATATTTGGTGT	MERS_9.2F	3981		TGACGCAAACTAGCTCTA	MERS_9.2R	6112
TAGCTGCCTTGTATCGTC	MERS_10.1F	5837		TATTTTCGAGTTCAATGGGG	MERS_9.1R	6147
AGCTGCCTTGTATCGTCT	MERS_10.2F	5838		AGCCGCATTACAATCAATGAG	MERS_10.1R	8291
GTGATTCTAGTGAAATCGCCA	MERS_11.2F	7939		CTGAAGCCGCATTACAATCAAT	MERS_10.2R	8295
CGTAAGGCGAGGCGATAA	MERS_11.1F	8054		CGACAGTCAACTTCAAAAGA	MERS_11.1R	10302
AACATACAGCGAGACTGG	MERS_12.1F	9947		GCTGGAGTGCTAGGGTTA	MERS_11.2R	10327
TGCGGTAGCATGACTCTTAA	MERS_12.2F	10083		CACTGCCTTATCCTTCTCAT	MERS_12.1R	12230
TATTGGCAGCAACAGACC	MERS_13.1F	11929		CATCGTTGTCGAGCTTCT	MERS_12.2R	12369
TTTGACACTCCTAGCGTAC	MERS_13.2F	12039		GGGTCCGAAACAAGTCTT	MERS_13.2R	14395
ACATGGTCAAGGCTGGTT	MERS_14.1F	13995		CCACAAGATACTACAAATGGC	MERS_13.1R	14445
ATGGTCAAGGCTGGTTTA	MERS_14.2F	13997		CAGGGGCATTACAAACGTA	MERS_14.1R	16367
ACAGGACACATGCTTGAC	MERS_15.1F	16097		GTAACGTCTGAAACGCCAC	MERS_14.2R	16389
AGTTCGCCTACCACTTTG	MERS_15.2F	16187		GTTCACACCAGTTGAAAATCC	MERS_15.1R	18349
CGAGTGATGAGCTTTGCG	MERS_16.1F	18105		GCAATGCCCGTTAACATG	MERS_15.2R	18411
TTTACCCGCAAATGTCCC	MERS_16.2F	18133		CCGCTGTGTTAGTCTCAGTA	MERS_16.2R	20423
TCTTGTAGTGACTTCCTACC	MERS_2.2F	20159		TATAACAGAACACACCGCCT	MERS_16.1R	20449
TACTACGTTAGGCGGTCT	MERS_2.1F	20290		GAGCAGTGGAGTTGTGACAAA	MERS_2.1R	22504
TGGCATTACACAAACTGCTCA	MERS_3.2F	22217		ACAACTGAGCCAGAAGGT	MERS_2.2R	22579
ATTTGCCACCTTGCCTGT	MERS_3.1F	22295		AGAACCTGTTGAGTAATGCC	MERS_3.1R	24424
TGATCCTGGTTATATGCAAGG	MERS_4.2F	24158		TAGAGCCTGTGCATTGTT	MERS_3.2R	24554
TTGGCAGCATAGCAGGTG	MERS_4.1F	24310		AGTAGGGATTGCCGTGTT	MERS_4.2R	26578
CAGGGTTACATTTTCAGACCC	MERS_5.2F	26263		GGAAAATCCCTGAACGAGAA	MERS_4.1R	26611
GTGGTATCTACGTTCGGG	MERS_5.1F	26290		GGCGGACTCCTGTAATTA	MERS_5.2R	28472
CAATCCTGAGACTAATTGCCTT	MERS_6.2F	28185		AGGAAACAGCACGAGGTG	MERS_5.1R	28599
TTGGTGGTACAACTGTCGT	MERS_7.2F	28220		CGCTGAGTGATGCTACCT	MERS_7.2R	29749
ACCTCAAAATGGCTGGCA	MERS_7.1F	28292		CAATCATTGGACCAGGCT	MERS_6.2R	29790
CATTTCGGTGCTTGTGAC	MERS_6.1F	28312		TGATTCCATTCTGTGCAAGAG	MERS_6.1R	29898
				TCTCTCTACAGCTACACACTTT	MERS_7.1R	29981

*Numbered according to EMC/2012 strain of MERS (GI:409052551)
