# Supplementary material for: Middle East Respiratory Syndrome Coronavirus Intra-Host Populations Are Characterized by Numerous High Frequency Variants
Source: PLoS One. 2016 Jan 20;11(1):e0146251. doi: 10.1371/journal.pone.0146251 (PMC4720378; doi:10.1371/journal.pone.0146251)
Supplement: S3 Table — (PDF) [file pone.0146251.s004.pdf]

Nt pos.	Data type	C1D1		C1D3		C1D5		C2D1		C2D3		C2D5		C3D1		C3D3		C3D5		SEED	
2360	Consensus	C	1.000	C	0.983	C	0.986	C	0.992	C	0.990	C	0.987	C	0.987	C	0.992	C	1.000	C	0.992
2360	iSNV	NA		T	0.017	T	0.014	T	0.008	T	0.010	T	0.013	T	0.013	T	0.009	NA		T	0.008
2360	Coverage	201		11393		1048		2160		1174		1588		1408		1883		948		2503	
2471	Consensus	C	1.000	C	1.000	C	1.000	C	0.991	C	0.995	C	1.000	C	1.000	C	1.000	C	1.000	C	0.994
2471	iSNV	NA		NA		NA		T	0.009	T	0.005	NA		NA		NA		NA		T	0.006
2471	Coverage	2602		11135		12483		24224		20768		16679		11100		22966		14657		27101	
4058	Consensus	C	0.996	C	0.998	C	1.000	0		C	1.000	C	1.000	C	1.000	0		C	1.000	C	1.000
4058	iSNV	A	0.004	A	0.002	NA		NA		NA		NA		NA		NA		NA		NA	
4058	Coverage	2184		2266		10839		NA		15997		10424		8968		NA		6245		966	
4069	Consensus	C	0.998	C	1.000	C	1.000	C	1.000	C	1.000	C	1.000	C	0.999	0		C	1.000	C	1.000
4069	iSNV	A	0.002	NA		NA		NA		NA		NA		A	0.001	NA		NA		NA	
4069	Coverage	1396		1499		8166		78		13853		9362		6677		NA		4825		1092	
4070	Consensus	C	0.995	C	1.000	C	1.000	C	1.000	C	1.000	C	1.000	C	0.999	C	1.000	C	1.000	C	1.000
4070	iSNV	A	0.005	NA		NA		NA		NA		NA		A	0.001	NA		NA		NA	
4070	Coverage	1354		1461		8086		88		13663		9293		6632		51		4735		1124	
6130	Consensus	C	0.995	0		C	1.000	C	1.000	C	1.000	C	1.000	C	1.000	C	1.000	C	0.997	C	1.000
6130	iSNV	A	0.005	NA		NA		NA		NA		NA		NA		NA		A	0.003	NA	
6130	Coverage	379		NA		2210		4915		6982		8258		2693		174		1654		11840	
8271	Consensus	C	1.000	C	1.000	C	1.000	C	1.000	C	1.000	C	0.996	C	1.000	C	1.000	C	1.000	C	0.998
8271	iSNV	NA		NA		NA		NA		NA		A	0.004	NA		NA		NA		A	0.002
8271	Coverage	342		75		9555		2070		7171		3186		4199		2076		3048		3600	
10085	Consensus	C	1.000	C	1.000	C	1.000	C	1.000	C	1.000	C	1.000	C	1.000	C	0.889	C	1.000	C	1.000
10085	iSNV	NA		NA		0		0		0		0		0		T	0.111	0		0	
10085	Coverage	71		75		5444		3779		6038		8038		6140		2562		3471		3535	
10086	Consensus	G	1.000	G	1.000	G	1.000	G	0.999	G	1.000	G	1.000	G	1.000	G	1.000	G	0.999	G	1.000
10086	iSNV	NA		NA		NA		A	0.001	NA		NA		NA		NA		NA		NA	
10086	Coverage	81		81		5476		3818		6130		8137		6165		2603		3490		3574	
12047	Consensus	T	1.000	T	1.000	T	1.000	T	0.985	T	1.000	T	1.000	T	0.988	T	1.000	T	1.000	T	1.000
12047	iSNV	NA		NA		NA		C	0.015	NA		NA		C	0.012	NA		NA		NA	
12047	Coverage	216		826		3229		4478		2205		3006		2607		4788		1851		5748	
18410	Consensus	G	0.997	G	1.000	G	1.000	G	0.999	G	1.000	G	1.000	G	1.000	G	1.000	G	1.000	G	1.000
18410	iSNV	T	0.003	NA		NA		T	0.001	NA		NA		NA		NA		NA		NA	
18410	Coverage	716		799		2625		6327		10718		9972		2451		2261		1592		10851	
24538	Consensus	0		0		A	0.710	A	0.803	A	1.000	G	0.698	A	0.944	A	0.845	A	0.956	A	0.966
24538	iSNV	NA		NA		G	0.290	G	0.197	NA		A	0.302	G	0.056	G	0.155	G	0.044	G	0.034
24538	Coverage	NA		NA		12906		2512		89		344		9493		994		5165		12338	
28312	Consensus	C	1.000	C	1.000	C	1.000	C	1.000	C	1.000	C	1.000	C	1.000	C	1.000	C	1.000	C	1.000
28312	iSNV	NA		NA		NA		NA		NA		NA		NA		NA		T	0.000	T	0.001
28312	Coverage	11380		19867		30869		60777		30453		74476		43013		27722		33031		47983	
28317	Consensus	C	0.997	C	1.000	C	1.000	C	0.999	C	1.000	C	1.000	C	0.999	C	1.000	C	0.999	C	1.000
28317	iSNV	T	0.003	NA		T	0.000	T	0.001	NA		T	0.000	NA		NA		T	0.001	T	0.000
28317	Coverage	32128		29100		33776		70871		39784		82368		47033		42984		36128		48149	
28318	Consensus	G	0.994	G	1.000	G	1.000	G	0.999	G	1.000	G	0.999	G	1.000	G	1.000	G	0.999	G	0.999
28318	iSNV	NA		NA		NA		A	0.000	NA		A	0.000	NA		NA		T	0.001	A	0.001
28318	Coverage	32068		29055		34689		73349		39678		85092		47934		42938		37225		48893	
28320	Consensus	T	1.000	T	1.000	T	1.000	T	1.000	T	1.000	T	1.000	T	1.000	T	1.000	T	1.000	T	1.000
28320	iSNV	NA		NA		NA		C	0.000	NA		NA		NA		NA		C	0.000	NA	
28320	Coverage	32329		29238		35830		75763		39600		88379		49415		43273		38830		49558	
28321	Consensus	G	1.000	G	1.000	G	1.000	G	0.999	G	1.000	G	1.000	G	1.000	G	1.000	G	1.000	G	1.000
28321	iSNV	NA		NA		NA		T	0.000	NA		NA		NA		NA		NA		NA	
28321	Coverage	32616		29474		37225		78758		40007		92151		50957		43681		40430		50528	
28322	Consensus	C	1.000	C	1.000	C	1.000	C	1.000	C	1.000	C	1.000	C	1.000	C	1.000	C	1.000	C	1.000
28322	iSNV	NA		NA		NA		NA		NA		T	0.000	T	0.000	NA		NA		NA	
28322	Coverage	32986		29870		38366		80260		41231		93601		51641		44446		43195		51681	

28324	Consensus	T	1.000	T	1.000	T	1.000	T	1.000	T	1.000	T	1.000	T	1.000	T	1.000	T	1.000	T	1.000
28324	iSNV	NA		NA		NA		C	0.000	NA		C	0.000	C	0.000	NA		NA		NA	
28324	Coverage	34134		31393		38828		81116		41156		94443		44109		44221		353714		33732	
28325	Consensus	G	1.000	G	0.996	G	0.999	G	1.000	G	1.000	G	1.000	G	1.000	G	1.000	G	1.000	G	1.000
28325	iSNV	NA		T	0.004	T	0.001	T	0.000	NA		T	0.000	NA		NA		NA		NA	
28325	Coverage	34451		31748		39102		81785		41326		95358		44299		44608		354766		34108	
28326	Consensus	T	1.000	T	1.000	T	1.000	T	1.000	T	0.998	T	0.998	T	0.996	T	0.992	T	0.990	T	1.000
28326	iSNV	NA		NA		NA		C	0.000	C	0.002	C	0.002	C	0.004	C	0.008	C	0.010	NA	
28326	Coverage	34493		31786		39205		82062		40343		95677		43338		44502		352441		34182	
28327	Consensus	G	0.998	G	1.000	G	1.000	G	1.000	G	1.000	G	1.000	G	1.000	G	1.000	G	1.000	G	1.000
28327	iSNV	T	0.002	NA		NA		NA		NA		T	0.000	NA		NA		T	0.000	NA	
28327	Coverage	34630		32114		39527		82706		40937		96651		43606		44788		352407		34179	
28328	Consensus	A	1.000	A	1.000	A	1.000	A	1.000	A	1.000	A	1.000	A	1.000	A	1.000	A	1.000	A	1.000
28328	iSNV	G	0.000	G	0.000	NA		NA		NA		NA		NA		NA		0		NA	
28328	Coverage	34648		32308		39694		82699		41409		96882		43611		44750		350702		34670	
28329	Consensus	C	0.999	C	1.000	C	1.000	C	1.000	C	1.000	C	1.000	C	1.000	C	1.000	C	1.000	C	1.000
28329	iSNV	NA		NA		NA		NA		NA		T	0.000	NA		NA		NA		NA	
28329	Coverage	34693		32385		39457		82294		41331		96181		43288		44483		348476		32894	
28458	Consensus	T	1.000	T	0.594	T	1.000	T	0.999	T	1.000	T	1.000	T	1.000	T	1.000	T	0.995	T	1.000
28458	iSNV	NA		C	0.406	NA		C	0.001	NA		NA		NA		NA		C	0.005	NA	
28458	Coverage	7593		15246		58715		74053		42135		53061		21253		27713		19050		86629	
28459	Consensus	T	1.000	T	0.977	T	1.000	T	1.000	T	1.000	T	1.000	T	1.000	T	0.999	T	1.000	T	1.000
28459	iSNV	NA		C	0.023	NA		NA		NA		NA		NA		C	0.001	NA		NA	
28459	Coverage	8290		15664		56055		71710		49106		53463		21624		31112		19330		83012	
28461	Consensus	C	1.000	C	1.000	C	1.000	C	0.999	C	0.996	C	0.999	C	0.999	C	0.998	C	0.989	C	0.999
28461	iSNV	NA		NA		NA		T	0.001	NA		T	0.001	T	0.001	NA		T	0.011	T	0.001
28461	Coverage	10721		18561		42719		56717		53628		47207		19702		34545		15801		61528	
28462	Consensus	A	1.000	A	1.000	A	0.999	A	1.000	A	1.000	A	1.000	A	1.000	A	1.000	A	1.000	A	1.000
28462	iSNV	NA		NA		NA		NA		NA		NA		NA		G	0.001	NA		NA	
28462	Coverage	11863		20017		17949		29296		64527		45175		15718		42315		9378		8597	
28463	Consensus	G	0.995	G	0.966	G	1.000	G	0.999	G	1.000	G	1.000	G	0.999	G	0.997	G	0.992	G	1.000
28463	iSNV	T	0.005	T	0.034	NA		T	0.001	T	0.000	T	0.000	NA		A	0.002	T	0.008	NA	
28463	Coverage	12864		21723		18154		29397		78491		45277		15945		51587		9155		8351	
28464	Consensus	G	0.638	G	0.865	G	0.995	G	0.998	G	0.999	G	0.999	G	0.999	G	0.997	G	0.980	G	1.000
28464	iSNV	T	0.362	T	0.100	T	0.005	NA		T	0.001	T	0.001	NA		NA		T	0.020	NA	
28464	Coverage	14170		22306		18203		29717		80761		45277		16409		52693		9714		8301	
28465	Consensus	A	1.000	A	0.977	A	1.000	A	0.999	A	1.000	A	1.000	A	1.000	A	0.999	A	1.000	A	1.000
28465	iSNV	NA		C	0.012	T	0.000	NA		NA		NA		NA		NA		NA		NA	
28465	Coverage	15230		23493		20012		34121		88581		51736		18633		58266		10812		8369	
28466	Consensus	G	1.000	G	0.920	G	0.996	G	0.997	G	0.999	G	0.999	G	0.998	G	0.997	G	0.965	G	0.999
28466	iSNV	NA		T	0.080	T	0.004	NA		T	0.001	NA		NA		NA		T	0.025	NA	
28466	Coverage	16366		24898		21032		36905		95117		55398		20492		63321		11891		8396	
28467	Consensus	T	1.000	T	0.986	T	0.996	T	0.999	T	1.000	T	0.999	T	0.999	T	0.999	T	0.994	T	1.000
28467	iSNV	NA		C	0.014	C	0.004	C	0.001	C	0.000	C	0.001	C	0.001	C	0.001	C	0.006	NA	
28467	Coverage	17266		25517		21255		37375		97409		55909		21161		65404		12399		8383	
28468	Consensus	C	0.997	C	1.000	C	0.999	C	0.999	C	1.000	C	1.000	C	1.000	C	1.000	C	1.000	C	1.000
28468	iSNV	A	0.003	NA		T	0.001	T	0.001	T	0.000	NA		NA		NA		NA		NA	
28468	Coverage	19125		26150		22963		41893		103559		61182		23633		72192		13801		8465	
28469	Consensus	C	0.994	C	1.000	C	1.000	C	0.999	C	1.000	C	0.997	C	0.999	C	0.999	C	0.995	C	1.000
28469	iSNV	A	0.006	NA		NA		NA		T	0.000	NA		NA		NA		T	0.005	NA	
28469	Coverage	20961		28694		27218		53684		116531		75491		29621		86416		16258		10625	
28470	Consensus	G	0.995	G	0.987	G	0.997	G	0.995	G	0.998	G	0.996	G	0.996	G	0.996	G	0.964	G	0.999
28470	iSNV	A	0.003	NA		A	0.002	A	0.003	A	0.001	C	0.002	A	0.003	NA		A	0.023	NA	
28470	Coverage	22635		30884		30272		58662		129261		82413		33041		97296		17910		11741	
28471	Consensus	C	0.998	C	0.980	C	0.999	C	0.998	C	0.997	C	0.999	C	0.999	C	0.998	C	0.994	C	1.000
28471	iSNV	NA		NA		NA		T	0.001	A	0.002	T	0.001	NA		T	0.001	NA		NA	

28471	Coverage	24048		34986		38539		81604		148762		107088		43019		121057		21782		13690	
28472	Consensus	C	0.995	C	1.000	C	1.000	C	1.000	C	1.000	C	1.000	C	1.000	C	1.000	C	1.000	C	1.000
28472	iSNV	A	0.005	NA		NA		A	0.000	NA		NA		A	0.000	NA		NA		NA	
28472	Coverage	26310		39482		50181		117312		171521		146681		58273		149377		25929		18287	
28585	Consensus	C	0.997	C	1.000	C	1.000	C	1.000	C	1.000	C	1.000	C	1.000	C	1.000	C	1.000	C	1.000
28585	iSNV	A	0.003	NA		NA		NA		NA		NA		NA		NA		NA		T	0.000
28585	Coverage	4237		1547		3018		7801		5722		2122		2106		3139		2428		41009	
28587	Consensus	C	0.996	C	0.993	C	0.990	C	0.996	C	0.950	C	0.996	C	1.000	C	1.000	C	1.000	C	0.993
28587	iSNV	NA		T	0.007	T	0.010	T	0.004	T	0.050	T	0.004	NA		NA		NA		T	0.007
28587	Coverage	6905		2716		4823		9759		7750		3658		3231		5087		3901		38098	
28597	Consensus	C	0.999	C	1.000	C	1.000	C	0.999	C	1.000	C	1.000	C	1.000	C	1.000	C	1.000	C	1.000
28597	iSNV	NA		NA		NA		T	0.001	NA		NA		NA		NA		NA		NA	
28597	Coverage	25069		10298		17338		23266		15711		11448		12582		18031		13995		35927	
29733	Consensus	G	1.000	G	1.000	G	1.000	G	1.000	G	0.997	G	0.999	G	1.000	G	0.999	G	0.996	G	0.995
29733	iSNV	NA		NA		NA		NA		NA		C	0.001	NA		C	0.001	C	0.004	NA	
29733	Coverage	123		370		1491		4392		1828		3206		2949		4045		2455		4381	
29734	Consensus	G	1.000	G	0.571	G	0.997	G	0.935	G	0.990	G	0.990	G	1.000	G	1.000	G	0.943	G	1.000
29734	iSNV	NA		T	0.429	C	0.003	NA		NA		NA		NA		NA		NA		NA	
29734	Coverage	105		364		1494		4326		1827		3188		2885		3980		2377		4303	
29735	Consensus	T	1.000	T	1.000	T	1.000	T	0.998	T	1.000	T	1.000	T	1.000	T	0.999	T	0.998	T	1.000
29735	iSNV	NA		NA		NA		A	0.002	NA		NA		NA		A	0.001	A	0.002	NA	
29735	Coverage	106		361		1501		4307		1835		3213		2823		3856		2293		4236	
29736	Consensus	A	1.000	A	1.000	A	1.000	A	0.999	A	0.997	A	1.000	A	0.989	A	0.996	A	0.989	A	0.921
29736	iSNV	NA		NA		NA		C	0.001	NA		NA		NA		NA		NA		C	0.079
29736	Coverage	98		358		1501		3686		1506		3078		2118		3132		1889		4150	
29737	Consensus	G	1.000	G	1.000	G	0.928	G	0.906	G	0.924	G	1.000	G	0.932	G	1.000	G	0.904	G	0.999
29737	iSNV	NA		NA		T	0.072	T	0.094	T	0.076	NA		T	0.063	NA		T	0.089	C	0.001
29737	Coverage	89		355		1523		3735		1485		3020		2022		3019		1794		4003	
29738	Consensus	C	1.000	C	0.994	C	1.000	C	1.000	C	0.997	C	0.962	C	0.989	C	1.000	C	1.000	C	0.957
29738	iSNV	NA		NA		NA		NA		G	0.003	NA		NA		NA		NA		NA	
29738	Coverage	73		343		1563		3885		1384		3009		2018		2873		1778		3967	
29739	Consensus	A	1.000	A	1.000	A	0.998	A	1.000	A	0.996	A	0.997	A	0.988	A	1.000	A	0.985	A	0.951
29739	iSNV	NA		NA		T	0.002	NA		NA		T	0.003	NA		NA		NA		NA	
29739	Coverage	64		333		1510		3714		1338		2908		1914		2768		1678		3741	
29740	Consensus	T	1.000	T	1.000	T	1.000	T	1.000	T	1.000	T	1.000	T	0.993	T	1.000	T	0.994	T	0.987
29740	iSNV	NA		NA		NA		NA		NA		NA		A	0.004	NA		G	0.003	NA	
29740	Coverage	62		333		1628		3930		1373		3001		1952		2892		1749		3832	
29741	Consensus	C	1.000	T	1.000	C	1.000	C	1.000	C	1.000	C	0.998	C	0.981	C	1.000	C	1.000	C	0.905
29741	iSNV	NA		0		NA		NA		NA		G	0.002	NA		NA		NA		T	0.095
29741	Coverage	61		327		1758		4183		1444		3164		2054		3025		1800		3948	
29742	Consensus	A	1.000	A	1.000	A	0.996	A	1.000	A	0.997	A	0.997	A	0.993	A	1.000	A	0.997	A	0.955
29742	iSNV	NA		NA		NA		NA		C	0.003	NA		NA		NA		NA		NA	
29742	Coverage	57		319		1781		4195		1413		3154		1974		2963		1737		3803	
29743	Consensus	C	1.000	C	1.000	C	0.995	C	1.000	C	0.974	C	0.959	C	0.974	C	1.000	C	1.000	C	0.921
29743	iSNV	NA		NA		A	0.005	NA		NA		NA		NA		NA		NA		T	0.072
29743	Coverage	54		316		1838		4338		1410		3188		1973		2956		1703		3754	
29744	Consensus	0		T	1.000	T	0.997	T	1.000	T	0.997	T	0.858	T	0.994	T	0.799	T	0.985	T	0.952
29744	iSNV	NA		NA		C	0.003	NA		NA		C	0.142	NA		A	0.201	NA		NA	
29744	Coverage	NA		307		1861		4293		1401		3074		1895		2873		1637		3594	
29745	Consensus	0		C	1.000	C	0.996	C	1.000	C	1.000	C	1.000	C	0.983	C	1.000	C	0.995	C	0.966
29745	iSNV	NA		NA		T	0.004	NA		NA		NA		NA		NA		NA		NA	
29745	Coverage	NA		295		1932		4622		1361		3143		1945		2811		1589		3570	
29746	Consensus	0		A	0.993	A	0.992	A	0.995	A	1.000	A	0.999	A	0.990	A	1.000	A	0.988	A	1.000
29746	iSNV	NA		NA		NA		NA		NA		T	0.001	NA		NA		NA		NA	
29746	Coverage	NA		286		1963		4652		1415		3174		1930		2872		1560		3509	
29747	Consensus	0		G	1.000	G	0.985	G	0.936	G	0.800	G	1.000	G	0.992	G	0.876	G	0.911	G	0.992

29747	iSNV	NA	NA		NA		T	0.064	T	0.190	NA		A	0.008	T	0.124	T	0.073	A	0.008
29747	Coverage	NA	284		1997		4751		1444		3182		1893		2918		1515		3424	
29748	Consensus	0	C	1.000	C	0.994	C	0.973	C	1.000	C	1.000	C	0.940	C	0.955	C	0.998	C	0.950
29748	iSNV	NA	NA		NA		NA		NA		NA		NA		NA		G	0.002	NA	
29748	Coverage	NA	281		2092		5002		1472		3270		1956		3007		1544		3450	
29749	Consensus	0	G	0.993	G	0.987	G	1.000	G	1.000	G	1.000	G	0.994	G	1.000	G	1.000	G	0.953
29749	iSNV	NA	NA		NA		NA		NA		NA		T	0.006	NA		NA		NA	
29749	Coverage	NA	270		2111		5301		1835		3348		2147		3426		1787		3397	
29782	Consensus	0	0		0		C	1.000	C	1.000	C	1.000	T	1.000	C	1.000	C	0.787	C	1.000
29782	iSNV	NA	NA		NA		NA		NA		0		0		NA		T	0.213	0	
29782	Coverage	NA	NA		NA		262		277		720		440		80		2584		588	
29783	Consensus	0	0		0		A	1.000	A	1.000	A	1.000	G	1.000	A	1.000	A	0.787	A	1.000
29783	iSNV	NA	NA		NA		NA		NA		0		0		NA		G	0.213	0	
29783	Coverage	NA	NA		NA		273		310		729		443		86		2588		585	
29784	Consensus	0	0		0		A	1.000	A	1.000	A	1.000	T	1.000	A	1.000	A	0.787	A	1.000
29784	iSNV	NA	NA		NA		NA		NA		0		0		NA		T	0.213	0	
29784	Coverage	NA	NA		NA		275		312		736		447		116		2594		588	
29788	Consensus	0	0		0		T	1.000	T	1.000	T	1.000	T	1.000	T	1.000	T	0.858	T	1.000
29788	iSNV	NA	NA		NA		NA		NA		NA		NA		NA		G	0.142	NA	
29788	Coverage	NA	NA		NA		281		317		768		451		136		3093		603	
